# Imeglimin Exerts Anti‐Tumor Activity in Multiple Myeloma Through Affecting Energy Metabolism and Downregulating IL‐16 Expression

**DOI:** 10.1002/cam4.71651

**Published:** 2026-03-04

**Authors:** Jifeng Jiang, Liang Ren, Yifeng Sun, Jing Li, Jiadai Xu, Aziguli Maihemaiti, Peng Liu

**Affiliations:** ^1^ Department of Hematology Huadong Hospital, Fudan University Shanghai China; ^2^ Department of Hematology Zhongshan Hospital, Fudan University Shanghai China; ^3^ Department of Hematology Shanghai Ninth People's Hospital, Shanghai JiaoTong University School of Medicine Shanghai China; ^4^ Department of Lymphoma The Affiliated Cancer Hospital of Xinjiang Medical University Urumqi China

**Keywords:** energy metabolism, IL‐16, imeglimin, multiple myeloma

## Abstract

**Background:**

Imeglimin (IME) is a novel oral anti‐diabetic agent with a similar chemical structure to metformin, which has shown broad‐spectrum anti‐tumor activity. However, the activity of imeglimin on tumor cells remains unclear. This study investigated the effects of IME on multiple myeloma (MM) cells and explored the underlying mechanisms.

**Methods:**

The effects of IME on MM cell proliferation were evaluated in vitro using MM cell lines and in MM cell‐derived xenograft (CDX) models. Seahorse metabolic analyses and RNA‐Seq were performed in IME‐treated and control MM cell lines. Single‐cell transcriptomic data were further analyzed to assess the role of IL‐16 in the bone marrow microenvironment.

**Results:**

IME inhibited MM cell proliferation and tumor growth in MM cell‐derived xenograft (CDX) models by inducing G1/G0 cell cycle arrest. IME suppressed oxidative phosphorylation and promoted glycolysis. IL‐16 mRNA expression was downregulated, and multiple cytokine–cytokine receptor interaction pathways were altered following IME treatment. The anti‐MM effect of IME was partly mediated by increased lactate production and decreased IL‐16 expression. Single‐cell transcriptomic data further demonstrated that IL‐16 plays an important role in the bone marrow microenvironment of MM.

**Conclusions:**

These findings suggest that IME may represent a novel approach for targeting IL‐16 and energy metabolism in the treatment of MM.

## 1 Introduction

Multiple myeloma (MM) is the second most common hematologic malignancy characterized by the clonal proliferation of plasma cells in the bone marrow. The introduction of proteasome inhibitors, immunomodulatory agents, and monoclonal antibodies has substantially improved patient outcomes; however, MM remains incurable. Due to the tumor heterogeneity of MM, some patients may be refractory to initial therapies or experience disease progression and relapse [1]. Consequently, there is a continued and urgent need for new therapeutic strategies, particularly for patients with relapsed or refractory MM.

Imeglimin (IME) is an oral hypoglycemic agent approved for the treatment of type 2 diabetes (T2DM) and is structurally related to metformin (MET), although its mechanisms of action differ substantially from those of MET [2]. MET has been extensively reported to exert broad‐spectrum anti‐tumor activity across multiple malignancies; however, whether IME possesses similar anti‐cancer properties remains largely unexplored. In metabolic studies using T2DM models, IME has been shown to modulate mitochondrial function, such as enhancing ATP generation and increasing NAD^+^ levels [3]. It has also been investigated that IME can regulate expression of cytokine [4]. These findings suggest that IME may affect cellular bioenergetics and inflammatory signaling, raising the possibility that it could exert previously unrecognized effects on tumor biology.

A variety of cytokines play critical roles in the pathogenesis and progression of MM, including IL‐6 and VEGF [5]. It has been previously demonstrated that IL‐16 is overexpressed in MM patients and is associated with poor prognosis. Knockdown of IL‐16 by small interfering RNA or blockade with anti‐IL‐16 antibody inhibits cell proliferation in MM cell lines [6]. Moreover, recent single‐cell RNA sequencing analyses also found that the activation of IL‐16 signaling pathways in MM rapid progressors [7], further highlighting its biological and clinical relevance. This study aimed to evaluate the anti‐myeloma effects of imeglimin, elucidate its impact on energy metabolism and IL‐16 expression and evaluate its potential role in MM therapy.

## 2 Material and Methods

### 2.1 Cell Culture, Reagents and Antibodies

Human myeloma cell lines (RPMI8226, H929 and MM.1S) were obtained from American Type Culture Collection (ATCC, VA, USA). All cell lines used in this study were authenticated by short tandem repeat (STR) profiling analysis and confirmed to be free of mycoplasma contamination. Cells were cultivated in RPMI1640 medium (Hyclone, UT, USA), supplemented with 10% fetal bovine serum (Gibco, NY, USA) at 37°C humidified atmosphere containing 5% CO_2_. IME (Imeglimin hydrochloride, HY‐14771A/CS‐2121; MedChemExpress, NJ, USA) was dissolved in phosphate‐buffered saline (PBS, Hyclone) as a stock solution of 100 mM and stored at −80°C. Recombinant human interleukin‐16 was from R&D Systems (MN, USA), which was processed into a stock solution of 50 μg/mL by sterile water. L(+)‐lactic acid was purchased from J&K Scientific (Beijing, China). The antibodies for Ki‐67, Cyclin D3 antibody (CCND3), CDK4, p27, and p21 were purchased from Cell Signaling Technology (CST, MA, USA).

### 2.2 Cell Viability and Proliferation Assays

Cells viability was assessed with Cell Counting Kit‐8 (CCK‐8, Biosharp, Guangzhou, China). Briefly, myeloma cells were seeded in 96‐well plates (10^4^/well) and incubated without or with IME at indicated contractions at incubators for 48 h, then 10 μL CCK‐8 reagent was added and the cells viability was measured based on the manufacturer's instruction after 2 h.

### 2.3 EdU Staining and Immunofluorescence

RPMI 8226 and MM.1S cell lines were treated with IME for 24 h. Samples were cultured with 10 μM 5‐ethynyl‐2′‐deoxyuridine (Thermo Fisher Scientific, NY, USA) for 2 h, fixed in 4% paraformaldehyde for 15 min, and then washed in PBS (Hyclone). The cells were subsequently permeabilized with 0.5% Triton X‐100 (Beyotime, Haimen, China) for 15 min. Finally, the cells were incubated with 200 μL of staining solution following the manufacturer's instructions. The nucleus was stained with DAPI (Beyotime). The results were visualized using a Nikon Eclipse CiL immunofluorescence microscope (Nikon, Tokyo, Japan) at 100× magnification.

### 2.4 Cell Line‐Derived Xenograft (CDX) Model

B‐NDG (NOD.CB17‐PrkdcscidIl2rgtm1/Bcgen) mice were purchased from Biocytogen (Beijing, China). The animal experiment has been granted ethical approval from the Ethics Committee of Fudan University‐affiliated Zhongshan Hospital. Five to eight weeks old female mice were used for tumor transplantation. 0.5 × 10^6^ cells/100 μL RPMI 8226 cells were implanted subcutaneously. Using the formula volume (mm^3^) = (length) × (width)^2^/2, the tumor volume was calculated every 3 days. When the tumor volume reached approximately 100 mm^3^, mice were randomly assigned to control and treatment groups based on similar average tumor volumes to ensure balanced distribution. Mice in the treatment group were gavaged with 200 mg/kg/day IME. Once the mouse with the smallest tumor burden first reached a tumor length of 1.5 cm, all mice in both groups were euthanized.

### 2.5 Immunohistochemistry

The mice tumor tissues were fixed with 4% paraformaldehyde and embedded in paraffin. 2 μm‐thick paraffin‐embedded sections were stained with hematoxylin and eosin (HE) and immunohistochemical staining. The sections were incubated with ki‐67 antibody (CST) overnight at 4°C. After three washings with PBS, the sections were incubated for 50 min at room temperature with horseradish peroxidase‐coupled secondary antibody. Dako REAL EnVision Detection System, Peroxidase/DAB+, Rabbit/Mouse (DAKO, Glostrup, Denmark) was used for detecting the sections. Images of the immunohistochemical sections were observed using a COIC XSP‐204 microscope (COIC, Chongqing, China) at 10× magnification.

### 2.6 Cell Cycle Assay

RPMI 8226 and MM.1S cells were selected for the cell‐cycle‐arrest experiments because they exhibited higher sensitivity and more consistent responses to IME in the CCK‐8 assays, making them suitable models for assessing IME‐induced cell‐cycle regulation. MM cells were starved for 24 h then incubated without or with IME at indicated concentrations for 48 h in 6‐wells plate. MM cells were harvested and fixed with pre‐cooled 75% ethanol at 4°C. The cell cycle was determined using the Cell Cycle Kit (BD Biosciences, CA, USA) following the manufacturer's instructions. Subsequently, the samples were then analyzed by flow cytometry.

### 2.7 Western Blot Analysis

MM cells were lysed in RIPA and quantified by a bicinchoninic acid assay (Beyotime). Protein extracts were separated on 10% sodium dodecyl sulfate‐polyacrylamide gel electrophoresis (SDS‐PAGE), then transferred to a polyvinylidene difluoride (PVDF, Millipor‐esigma, MA, USA) membrane, after blocking with 5% non‐fat milk, incubating with the appropriate primary antibody followed by HRP‐conjugated secondary antibodies incubation. Last, protein signals were then detected with an ECF Western blotting kit.

### 2.8 Measurement of Oxygen Consumption Rate (OCR) and Extracellular Acidification Rate (ECAR)

The extracellular acidification rate (ECAR) and cellular oxygen consumption rate (OCR) were measured using a Seahorse XF96 Extracellular Flux Bioanalyzer (Seahorse Bioscience, MA, USA). RPMI‐8226 and MM.1S cell lines were treated with IME (1 mM) for 24 h. Cells were collected and 7.5–15 × 10^4^ cells/well seeded in XFp Cell Culture miniplates. Cell Mito Stress Test kit (Agilent, CA, USA) was used to measure the OCR. Oligomycin (1 μM) was injected following basal OCR measurements followed by injection of FCCP (1 μM), and finally by injection of a mixture of rotenone (1 μM) and antimycin A (1 μM). For ECAR, using the Seahorse XF Glycolysis Stress Test Kit, sequentially injected glucose (10 mM), Oligomycin A (1 μM), and 2‐deoxyglucose (2‐DG; inhibitor of glycolysis; final concentration, 50 mM) into each well.

### 2.9 RNA‐Sequence

Myeloma cells (RPMI 8226) treated with or without IME (5 mM) were harvested for RNA‐seq. The whole process of RNA‐seq was performed by Shanghai OE Biotech. Genes picked by cutoff with |log_2_(FoldChange)| > 1.5 and *q* < 0.01 were assigned as differentially significantly expressed.

### 2.10 Measurement of Lactic Acid in Cell Culture Supernatants and Serum of Mice

Lactic acid level in cell culture supernatants was determined by Lactic Acid assay kit (Nanjing Jiancheng Bioengineering Institute, Nanjing, China) according to the manufacturer's protocol. Lactic acid in serum of mice was measured by Hitachi 3500 automatic analyzer (Hitachi, Tokyo, Japan).

### 2.11 Quantitative Real‐Time PCR Analysis

Total RNA was extracted from treated myeloma cells using the RNA Purification Kit (EZBioscience, MN, USA) according to the manufacturer's protocol. Total RNA (500 ng) was reverse transcribed using the Reverse Transcription kit (Takara Biomedical Technology, Beijing, China). Each cDNA sample was amplified by SYBR qPCR MasterMix (Takara Biomedical Technology). Primers for β‐actin and IL‐16 quantification were obtained from Generay (Shanghai, China). β‐actin: Forward: AAGGAGCCCCACGAGAAAAAT, Reverse: ACCGAACTTGCATTGATTCCAG; IL‐16: Forward: TTGGACACAGGGTTCTCGCTCA, Reverse: AGCAGGGAGATAACGGACTGAC.

### 2.12 Single Cell RNA‐Sequence Analysis

Four healthy subjects from GEO database referred to as control sample (accession number GSE223060), five newly diagnosed MM samples were from CNGB Sequence Archive (CNSA) of China National GeneBank DataBase (CNGBdb, accession number CNP0002345). Single‐cell RNA sequencing was performed using bone marrow mononuclear cells collected from MM patients. The experimental workflow followed the procedures described by Li et al. [8]. Briefly, freshly isolated bone marrow aspirates were processed to obtain mononuclear cells, which were loaded onto the 10× Genomics Chromium platform for droplet‐based single‐cell encapsulation and barcoding. cDNA library preparation and sequencing were performed according to the manufacturer's protocol. Downstream bioinformatic analyses methodology can be found in our previously published paper by Ren et al. [9]. This study was performed in line with the principles of the Declaration of Helsinki and approved by the Clinical Research Ethics Committee of Fudan University‐affiliated Zhongshan Hospital. Informed consent was obtained from all patients for being included in the study.

### 2.13 Statistical Analysis

Each vitro experiment was performed in triplicate, and the results expressed as the mean ± standard deviation (SD). A two‐sided *p* values < 0.05 were considered statistically significant. The version 8.0.2 GraphPad prism software (San Diego, CA, USA) and The R software version 4.0.5 were used for statistical analyses.

## 3 Results

### 3.1 IME Exerts Anti‐MM Activity In Vitro and In Vivo

**FIGURE 1:**
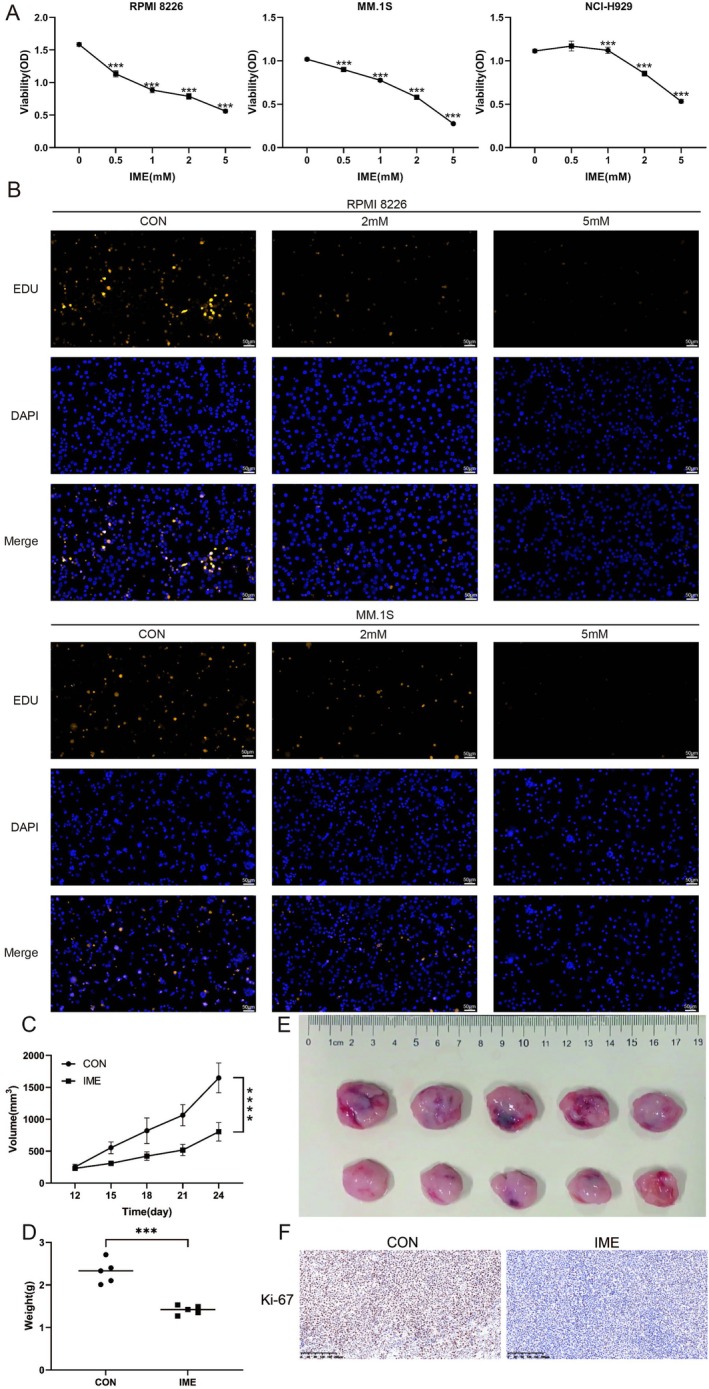
IME inhibits multiple myeloma cell proliferation and tumor growth in vivo. (A) RPMI 8226, MM.1S, and NCI‐H929 cells were treated with 0.5, 1, 2, or 5 mM IME for 48 h, and cell viability was assessed by the CCK‐8 assay (*n* = 3). (B) RPMI 8226 and MM.1S cells were subjected to EdU staining after treatment with 2 or 5 mM IME for 48 h (scale bar: 50 μm). (C) Tumor volume in the CDX model was measured over time after IME treatment (*n* = 5). (D, E) Tumor weight and tumor volume at the experimental endpoint. (F) Immunohistochemical staining of Ki‐67 expression in mice tumor tissues (scale bar: 50 μm). Data are presented as mean ± SD, with ***p < 0.005, ****p < 0.001.

RPMI 8226, MM.1S and NCI‐H929 multiple myeloma cells were treated with increasing concentrations of IME for 48 h. CCK‐8 assays demonstrated a dose‐dependent reduction in cell viability, with statistically significant differences compared with the control group (*p* < 0.005, Figure 1A). EdU fluorescence staining further confirmed the inhibitory effect of IME on cell proliferation (Figure 1B). The in vivo efficacy of IME was further evaluated in CDX model (*n* = 5). IME‐treated tumors displayed a significantly slower growth trend compared with controls over the entire monitoring period (*p* < 0.001, Figure 1C), whereas final tumor volumes and weights at the experimental endpoint were also significantly reduced (*p* < 0.005, Figure 1D,E). IHC staining showed that the expression of Ki‐67 in tumor tissues of IME group was decreased (Figure 1F).

### 3.2 IME Induces G1/G0 Cell Cycle Arrest and Regulates the Expression of Cell Cycle‐Related Proteins in MM Cells

**FIGURE 2:**
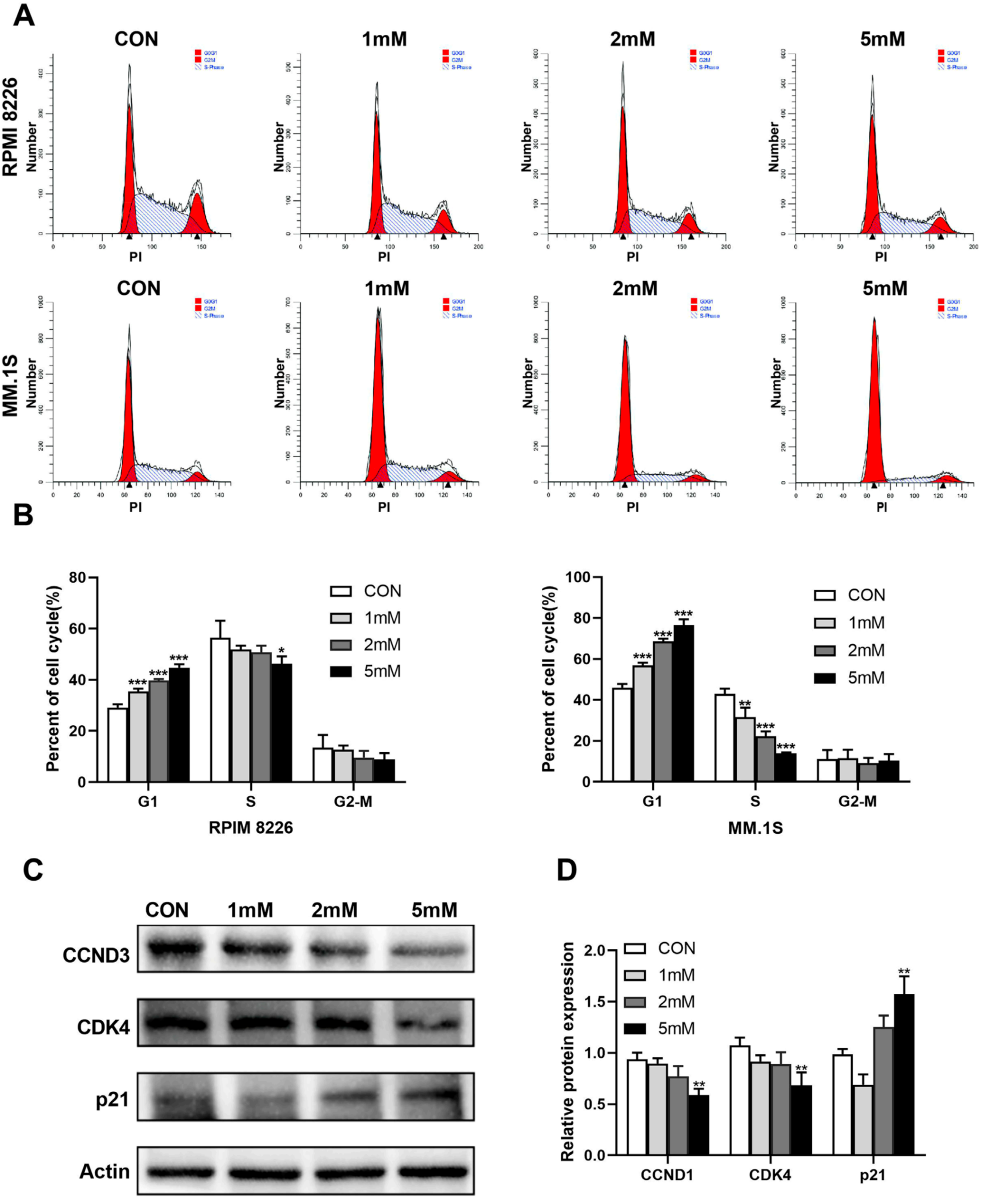
IME induces G1/G0 cell cycle arrest and regulates the expression of cell cycle‐related proteins in MM cells. (A) Following the treatment with 1, 2 or 5 mM IME for 48 h, cell cycle phases was then detected by PI staining using flow cytometry. Quantitative cell‐cycle data were obtained from three independent biological experiments, and the plots shown represent typical reproducible results. (B) Quantitation of cells at different cell cycle phases (*n* = 3). (C) Western blot analysis of cell cycle regulatory proteins in RPMI‐8226 after treatment with IME. (D) Quantification of Western blot. Data are presented as mean ± SD, with *p < 0.05, **p < 0.01, ***p < 0.005.

Using flow cytometry, we examined the proportion of each cell cycle phase in RPMI 8226 and MM.1S cell line treated with different concentrations of IME. The proportion of MM cell lines in G1/G0 phase increased (*n* = 3, *p* < 0.005) and S phase decreased with the increase of IME concentration. These results were consistent with the CCK‐8 assays, as MM.1S was more sensitive to IME than RPMI 8226 cell line (Figure 2A,B). To further confirm that the effects of IME on cell cycle, we performed Western blot to detect cell cycle protein expression levels. The results demonstrated a dose‐dependent effect of IME on cell‐cycle regulators, with CCND1 and CDK4 decreased and p21 increased, and significant differences observed at 5 mM compared with controls (*p* < 0.01) (Figure 2C,D). These findings suggest that IME may be associated with G1 arrest in MM cells through modulation of CCND1, CDK4, and p21.

### 3.3 IME Inhibites Oxidative Phosphorylation (OXPHOS) and Promotes Glycolysis in MM Cells

**FIGURE 3:**
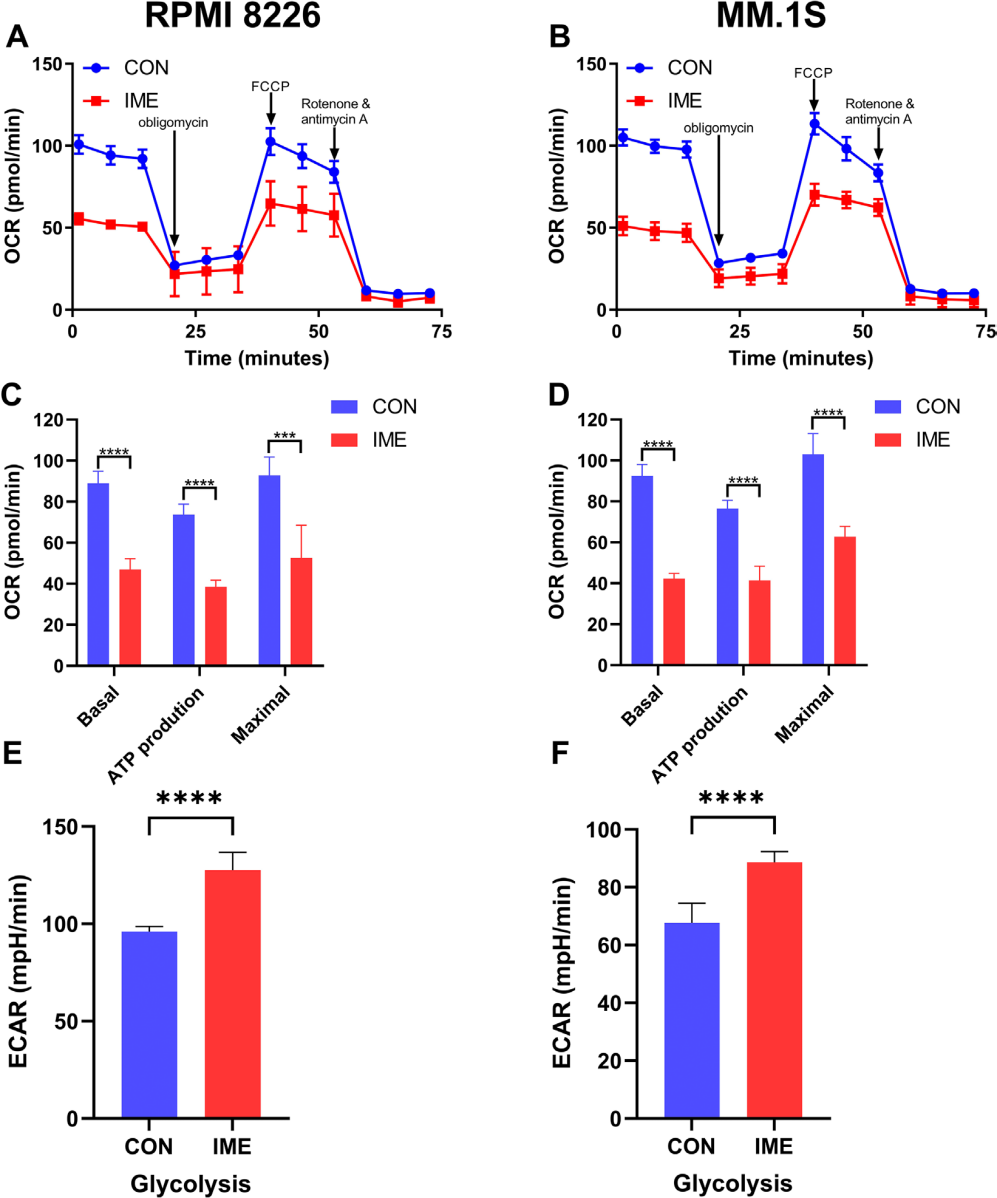
IME inhibits oxidative phosphorylation (OXPHOS) and promotes glycolysis in MM cells. (A–D) Oxygen consumption rate (OCR) measurements in MM cells treated with 1 mM IME for 24 h, showing changes in basal respiration, ATP‐linked OCR, and maximal respiration (*n* = 3). (E, F) Extracellular acidification rate (ECAR) measurements following IME treatment (*n* = 3). Data are presented as mean ± SD, with ***p < 0.005, ****p < 0.001.

We hypothesized that the inhibitory effects of IME on MM cell proliferation and cell‐cycle progression may be associated with altered energy metabolism. Using the Seahorse XF analyzer, we evaluated mitochondrial respiration and glycolytic activity in MM cells. IME treatment significantly reduced basal OCR, ATP‐linked OCR and maximal OCR in both RPMI 8226 and MM.1S cells (*p* < 0.005, Figure 3A–D). In addition, IME treatment significantly enhanced glycolytic activity, as reflected by increased ECAR (*p* < 0.001, Figure 3E,F). These findings indicate that IME suppresses OXPHOS while promoting glycolysis.

### 3.4 IME Regulates IL‐16 Expression and Cytokine‐Cytokine Receptor Pathway

**FIGURE 4:**
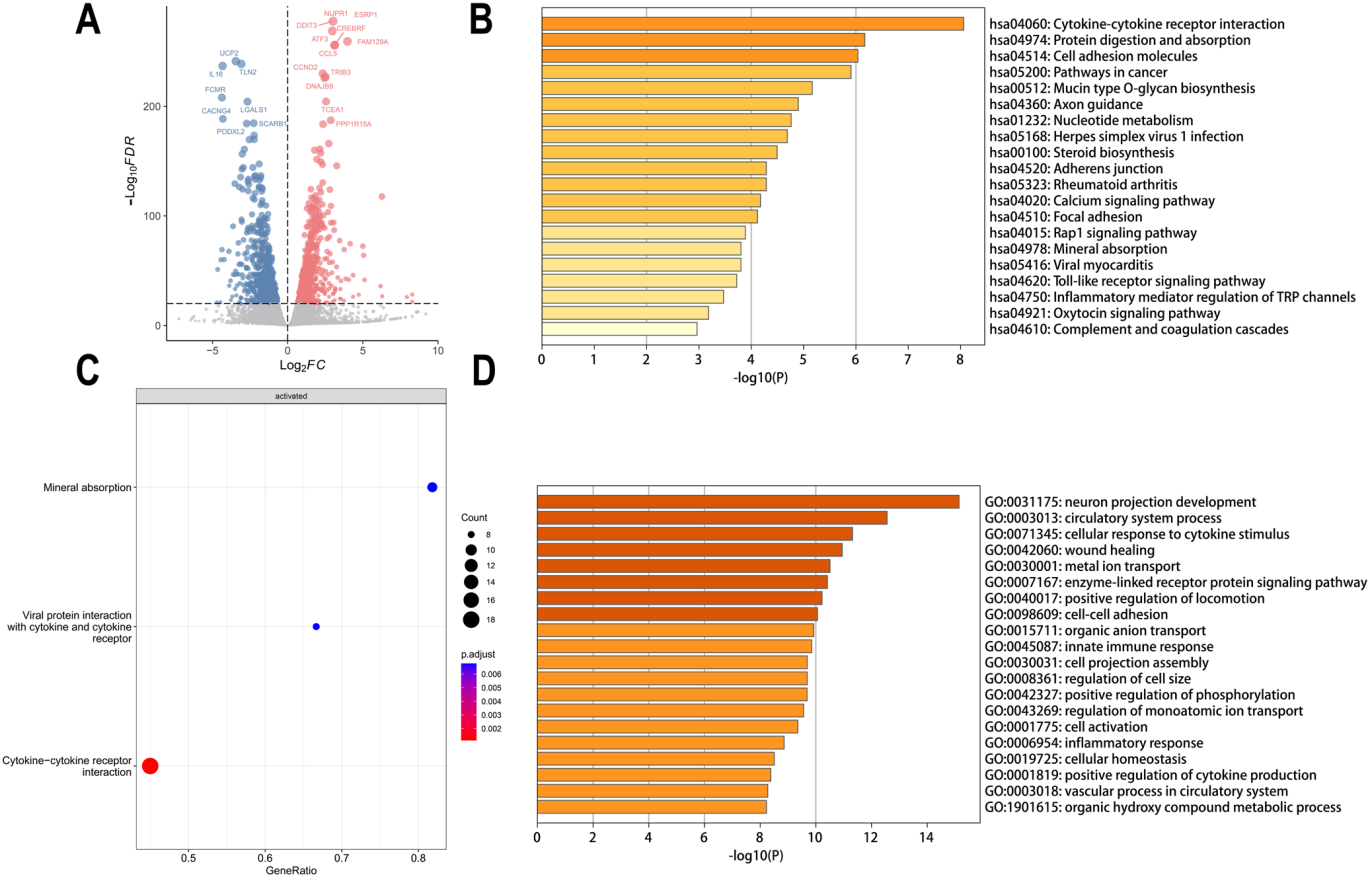
IME regulates IL‐16 expression and cytokine‐cytokine receptor pathway. (A) Volcano plot showing the distribution of differentially expressed genes (DEGs) following IME treatment. (B) KEGG analysis of DEGs. (C) GSEA analysis of DEGs. (D) GO analysis of DEGs.

To further elucidate the mechanism by which IME inhibited the proliferation of MM cell lines, we performed RNA‐Seq on IME‐treated cells and control cells. The results suggested that a variety of genes were differentially expressed in IME‐treated MM cell lines (*n* = 3, Figure 4A). Sorting of differential expression genes as described in the method section, the top 3 upregulated genes by *p*‐value included ESRP1, NUPR1 and DDIT3. The top 3 downregulated genes included UCP2, TLN2 and IL‐16. We conducted enrichment analysis including GO, KEGG and GSEA three approaches, and revealed that these genes were involved in pathways including cytokine‐ pathway (Figure 4B–D). The most significantly enriched pathways were cytokine−cytokine receptor interaction in both KEGG and GSEA analyses and the third most significant pathway, cellular response to cytokine stimulus, in GO analyses. Taken together the above results, we consider that the downregulation of IL‐16 could partly explain the mechanism responsible for the anti‐MM effect of IME.

### 3.5 High Concentrations of Extracellular Lactate Inhibites IL‐16 Expression and Recombinant IL‐16 Could Reverse the Anti‐Proliferative Effect of IME

**FIGURE 5:**
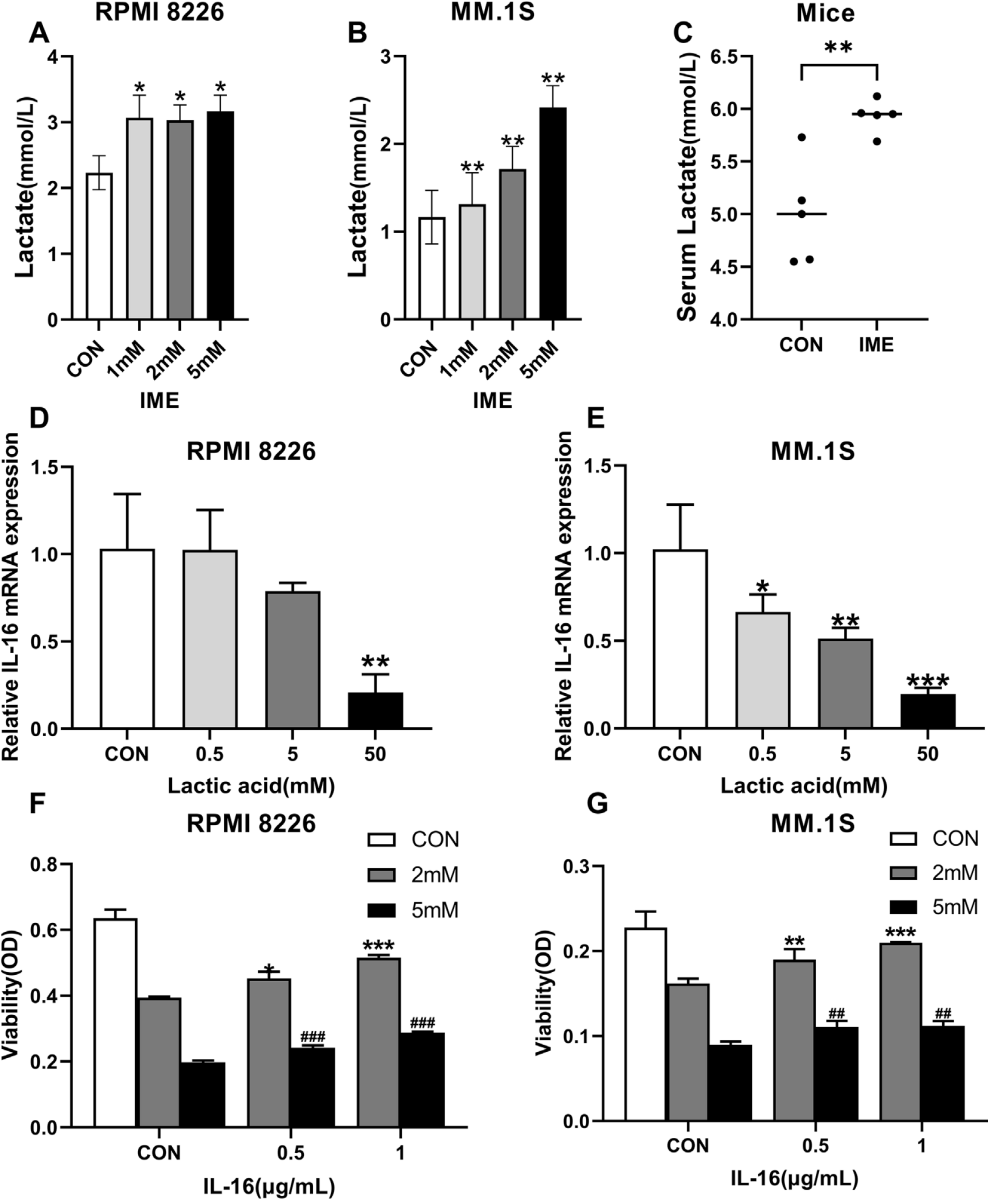
High concentrations of extracellular lactic acid inhibit IL‐16 expression and recombinant IL‐16 could reverse the anti‐proliferative effect of IME. (A, B) Lactate levels in cell culture supernatants of RPMI‐8226 and MM.1S cells. (C) Lactate levels in mouse serum after IME treatment. (D, E) IL‐16 mRNA expression in RPMI‐8226 and MM.1S cells treated with different concentrations of lactic acid. (F, G) Recombinant IL‐16 (0.5 or 1 μg/mL) was added to the culture medium before RPMI‐8226 and MM.1S cells were treated with 2 or 5 mM IME for 48 h. Cell viability was assessed by CCK‐8 assay. Data are presented as mean ± SD, with *p < 0.05, **p < 0.01, ***p < 0.005, ^##^ *p* < 0.01, ^###^ *p* < 0.005.

It has been reported that lactate can inhibit the expression of inflammatory cytokines [10]. We observed that lactate levels were significantly increased in the cell culture supernatants in cell line (*n* = 3, *p* < 0.05) and mouse serum (*n* = 5, *p* < 0.01) after IME treatment (Figure 5A–C). Based on these findings, we hypothesized that elevated lactate levels might contribute to the downregulation of IL‐16. Consistent with this hypothesis, culturing MM cells in media supplemented with exogenous lactate resulted in a reduction in IL‐16 mRNA expression (Figure 5D,E). To further determine whether reduced IL‐16 expression mediates the anti‐MM activity of IME, we conducted rescue experiments using recombinant IL‐16. RPMI‐8226 and MM.1S cells were pretreated with recombinant IL‐16 before exposure to IME. CCK‐8 assays demonstrated that recombinant IL‐16 partially restored cell viability in a dose‐dependent manner. Treatment with 0.5 μg/mL recombinant IL‐16 produced a modest but statistically significant increase in viability (*p* < 0.05), whereas the 1 μg/mL dose provided a more substantial rescue effect (*p* < 0.005, Figure 5F,G). Collectively, these findings indicate that the anti‐proliferative effects of IME are at least partially mediated through increased lactate production and the subsequent suppression of IL‐16 expression.

### 3.6 IL‐16 Plays an Important Role Within the Bone Marrow (BM) Microenvironment of MM.

**FIGURE 6:**
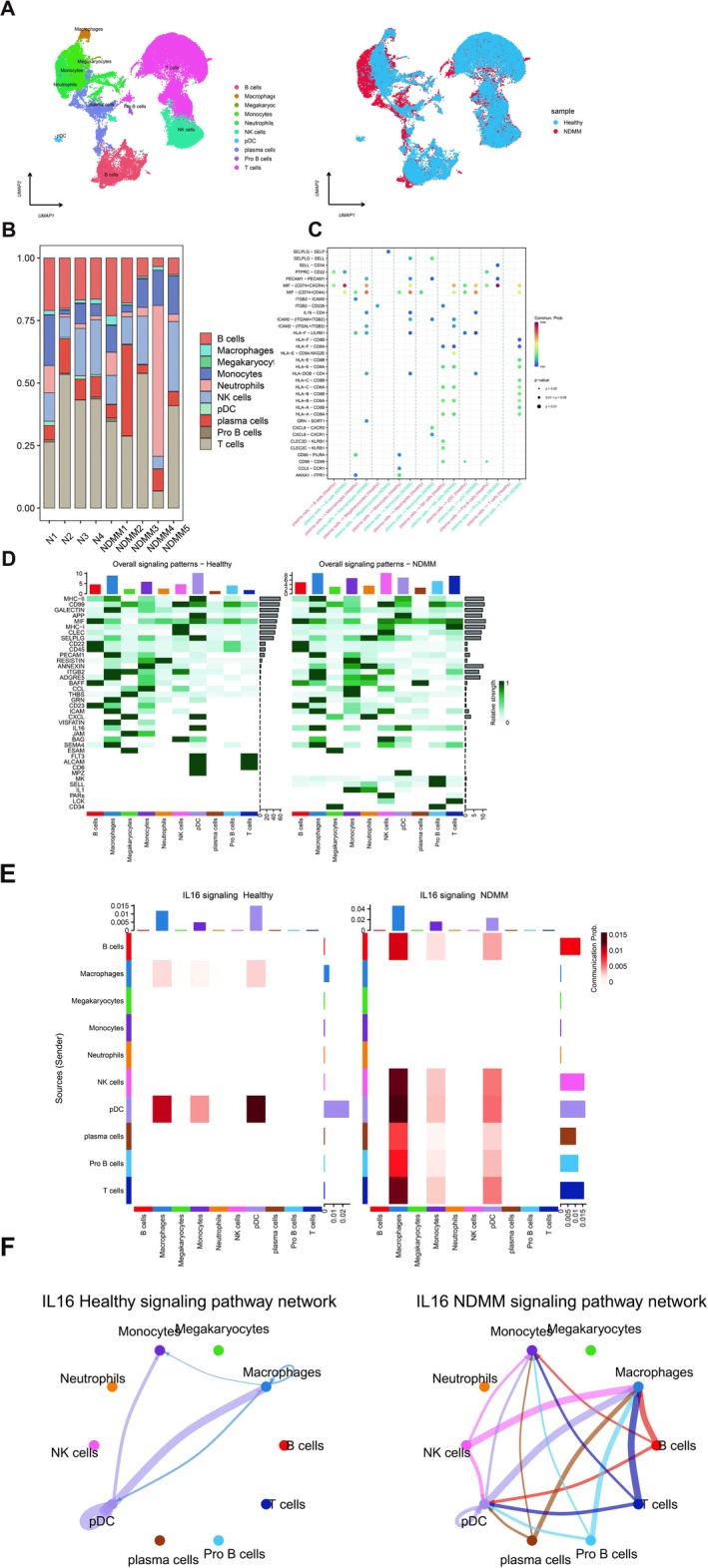
IL‐16 plays an important role within the bone marrow microenvironment of MM. (A) UMAP plot showing identification of 45,843 single cells colored by cell types (left) and cell origins from MM or healthy donors by color (right). (B) Cell type proportion of all samples. (C) Ligand–receptor interactions between cell types in MM and healthy samples. (D) Overall signaling patterns in MM and healthy samples. (E) IL16‐related signaling pathway networks in MM and healthy samples. (F) Ligand–receptor pairs in MM and healthy samples.

To explore the function of IL‐16 in the pathogenesis of MM, we analyzed single‐cell data from GEO database and CNGBdb database. 9 BM samples (5 MM patients and 4 healthy donors) were subject to single cell RNA‐sequence. After quality control, 45,843 cells were grouped into 10 cell types based on known gene markers (Figure 6A). These cell types were distributed at varying proportions across these samples, unveiling inter‐tumoral heterogeneity in cellular compositions within MM (Figure 6B). The comprehensive analysis of cell type interactions revealed a heightened level of interplay in the context of MM, as evidenced by both an increased quantity and enhanced interaction strength in MM compared to that observed in healthy subjects (Figure 6C). IL‐16 signal pathways network were more active on MM BM ecosystems compared to healthy donors (Figure 6D). The macrophages stand out prominently among these entities. Upon the introduction of MM cells, macrophages augmented their interactions with diverse immune cell populations via the IL‐16‐mediated signaling pathway (Figure 6E). We found an increased signaling of IL‐16‐CD4 in MM between plasma cells and macrophages or monocytes (Figure 6F).

## 4 Discussion and Conclusion

MM remains currently an incurable hematologic malignancy, searching for agents with different mechanisms of action is of great significance. As a classic medication for the treatment of T2DM, MET exerted a variety of anti‐tumor effects and reduced the risk of development and progression in various malignant tumor types [11, 12]. Previous study has demonstrated that taking MET was associated with a decreased risk of progression of MGUS to multiple myeloma [13]. MET also displayed anti‐MM activity through multiple mechanisms in vitro and in vivo [14, 15, 16]. IME as a novel derivative of MET, has been approved for treatment of T2DM in Japan [17]. However, IME and MET differ in several biological effects and underlying mechanisms of action [2]. Whether IME has a similar anti‐tumor activity has not been reported yet.

In this study, we observed that IME inhibited the proliferation of MM cells and suppressed tumor growth in a MM CDX model. The expression of Ki‐67 in tumor tissues from IME‐treated mice was significantly decreased. Together, these results showed that IME displays antitumor activity in vitro and vivo (Figure 1). We also found IME induced G0/G1 cell cycle arrest in a dose‐dependent manner (Figure 2). Wang et al. reported that MET induces autophagy and G0/G1 phase cell cycle arrest in myeloma, with this effect being associated with activation of the AMPK/mTORC1 and mTORC2 pathways [16]. In our study, the IC50 value of IME in RPMI‐8226 cells was slightly lower than that reported for MET in the same cell line, suggesting that MM cells may exhibit greater sensitivity to IME [16]. Recently, other research illustrated that the sensitivity of prostate cancer cells to MET was related to cell cycle reactivation and metabolic reprogramming [18]. This finding indicates MET affects cellular energy metabolism and cell cycle‐related pathways in cancer cells; a similar mode of action can be expected for IME.

To assess whether the effect of IME was related to its regulation of cell energy metabolism, we analyzed OXPHOS and glycolysis in MM cells. The results showed that OXPHOS was inhibited by IME, whereas glycolysis was increased (Figure 3). It has been reported that both IME and MET have an inhibitory effect on OXPHOS [19, 20]. According to the Warburg effect, many cancers rely predominantly on glycolysis for energy production. However, in certain malignancies, including leukemias and lymphomas, OXPHOS can be upregulated and play an important role in supporting tumor cell proliferation [21, 22]. OXPHOS is also increased in MM, and its activation has been shown to promote tumorigenesis [23]. MET as an OXPHOS inhibitor has been reported to induce cell cycle arrest in pancreatic cancer [24]. Based on these results, we suggest that the anti‐MM effect of IME was related to the inhibition of OXPHOS. However, Bloedjes et al. [25] observed that the survival and proliferation of MM rely primarily on glycolysis. Therefore, the direct effect of elevated glycolysis cannot explain the inhibition of MM cell proliferation in our study. We hypothesize that the enhanced glycolysis can lead to changes in gene expression and signaling pathways that indirectly affect MM cell proliferation.

Cytokines play essential roles in the pathogenesis of MM [5]. IL‐6 is the most important growth factor in MM, promoting MM cell survival, homing, bone destruction and drug resistance [26, 27]. VEGF is another cytokine that has a significant impact on MM for its role in the survival, proliferation, and diffusion of tumor cells [28]. However, the therapeutic strategies using monoclonal antibodies targeting cytokines have failed in MM [28, 29]. Intriguingly, MET has been shown to inhibit IL‐6 signaling by downregulating IL‐6R expression on MM cells [30]. This sheds new light on targeting cytokines and their signaling pathways. Our RNA‐Seq data indicated that mRNA expression of IL‐16 in MM cells was decreased following the IME treatment (Figure 4A). Enrichment analysis also showed that various differentially expressed genes involved in the cytokine−cytokine receptor pathway were regulated by IME (Figure 4B–D). In the context of its antidiabetic activity, IME has been shown to improve mitochondrial and endoplasmic reticulum function, which may be associated with reduced expression of inflammatory cytokines [4]. However, there are few reports on the expression of specific cytokines regulated by IME. Currently, one study reported that IME suppresses proinflammatory cytokines production of IL‐1β in high‐glucose‐stimulated mouse microglia [31]. Our RNA‐Seq data also provides some information on cytokines expression regulated by IME.

It has been found that lactic acid production has a regulatory role on cytokines secreted by immune cells [32]. The glycolysis promoted by IME led to an increased production of lactic acid (Figure 5A–C). Therefore, we speculate that the expression of IL‐16 in MM cells could be influenced by the level of extracellular lactic acid. Consistent with our expectation, IL‐16 mRNA levels were reduced following lactic acid treatment, in a concentration‐dependent manner (Figure 5D,E). Brand et al. [10] observed that lactic acid suppresses IL‐2 and IFN‐γ expression in CD8+ T cells and NK cells and promotes apoptosis. The downregulation of cytokines' expression was attributed to impaired NFAT activation [10]. IL‐16 exerts a direct regulatory effect on MM cells. Knockdown of IL‐16 inhibits the proliferation of MM cells, and this effect can be rescued by the addition of recombinant IL‐16 peptide [33]. Using recombinant IL‐16, we found that the suppression of cell proliferation induced by IME was reversed (Figure 5F,G). These results indicate that the inhibitory effects of IME on proliferation and G0/G1 arrest were partially mediated by the downregulation of IL‐16. However, the addition of exogenous IL‐16 did not further enhance MM cell proliferation (Supporting Information). This may be due to the pro‐proliferative effects of IL‐16 reaching a saturation level in MM cells. Based on the above research, we suggest that IME inhibited IL‐16 expression by increasing glycolysis and lactate production.

IL‐16 was initially identified as a T‐cell growth factor and has been implicated in infections, autoimmune diseases, and cancers [33, 34]. Activation of IL‐16 signaling was associated with the progression of multiple tumors [33]. Recently, Guan et al. [35] reported that IL‐16 regulates the pro‐tumor microenvironment in the activated B cell‐like subtype of diffuse large B‐cell lymphoma. IL‐16 was also significantly overexpressed in MM bone marrow samples compared with healthy donors, and IL‐16 signaling pathways were activated in the MM rapid progressors [6, 7]. The analysis of single‐cell RNA sequencing data revealed that IL‐16 signaling pathways were enhanced in MM patients and that various immune cells, particularly macrophages, interact with MM cells through IL‐16 related signaling networks (Figure 6). These findings demonstrate that IL‐16 plays an important role in shaping the bone marrow immune microenvironment in MM patients.

This study has several limitations. First, although IME reduced IL‐16 expression and inhibited MM cell proliferation, the downstream signaling pathways linking IL‐16 to cell‐cycle regulation were not fully delineated, and IL‐16 overexpression or knockdown experiments were not performed. These approaches will be necessary to clarify the mechanistic role of IL‐16 more directly. Second, although the in vivo dose of IME was selected based on previously reported safe ranges, our safety evaluation was limited to monitoring body weight and did not include serum biochemical or hematological assessments. These mechanistic and toxicological aspects will be further addressed in subsequent studies.

In summary, our study revealed that IME exerts an anti‐proliferative effect in MM cells by inducing G0/G1 cell cycle arrest through modulating energy metabolism and downregulating IL‐16 expression. In addition, IME regulates other cytokines' expression and associated signaling pathways. IL‐16 plays an important role in the immune microenvironment of bone marrow in MM patients. These findings indicate that IME may represent a novel approach for targeting energy metabolism and IL‐16 for the treatment of MM and other IL‐16‐mediated disorders.

## Author Contributions

Peng Liu contributed to the conception and design of the study and revised the manuscript. Jifeng Jiang, Liang Ren, and Yifeng Sun conducted the experiments, analyzed the data, and wrote the manuscript. Jing Li and Jiadai Xu collected the patient specimens and performed single‐cell RNA sequencing experiments. Aziguli maihemaiti also performed the experiments.

## Ethics Statement

This study was approved by the Clinical Research Ethics Committee of Fudan University‐affiliated Zhongshan Hospital. Informed consent was obtained from all patients for being included in the study.

## Conflicts of Interest

The authors declare no conflicts of interest.

## Supporting information


**Figure S1:** cam471651‐sup‐0001‐FigureS1.jpg.

**Figure S2:** cam471651‐sup‐0002‐FigureS2.jpg.

**Figure S3:** cam471651‐sup‐0003‐FigureS3.jpg.

**Data S1:** cam471651‐sup‐0004‐Supinfo.zip.

## Data Availability

The data that support the findings of this study are available from the corresponding author upon reasonable request.
