# Supplementary figures and images for: Imeglimin Exerts Anti‐Tumor Activity in Multiple Myeloma Through Affecting Energy Metabolism and Downregulating IL‐16 Expression
Source: Cancer Med. 2026 Mar 4;15(3):e71651. doi: 10.1002/cam4.71651 (PMC12960283; doi:10.1002/cam4.71651)

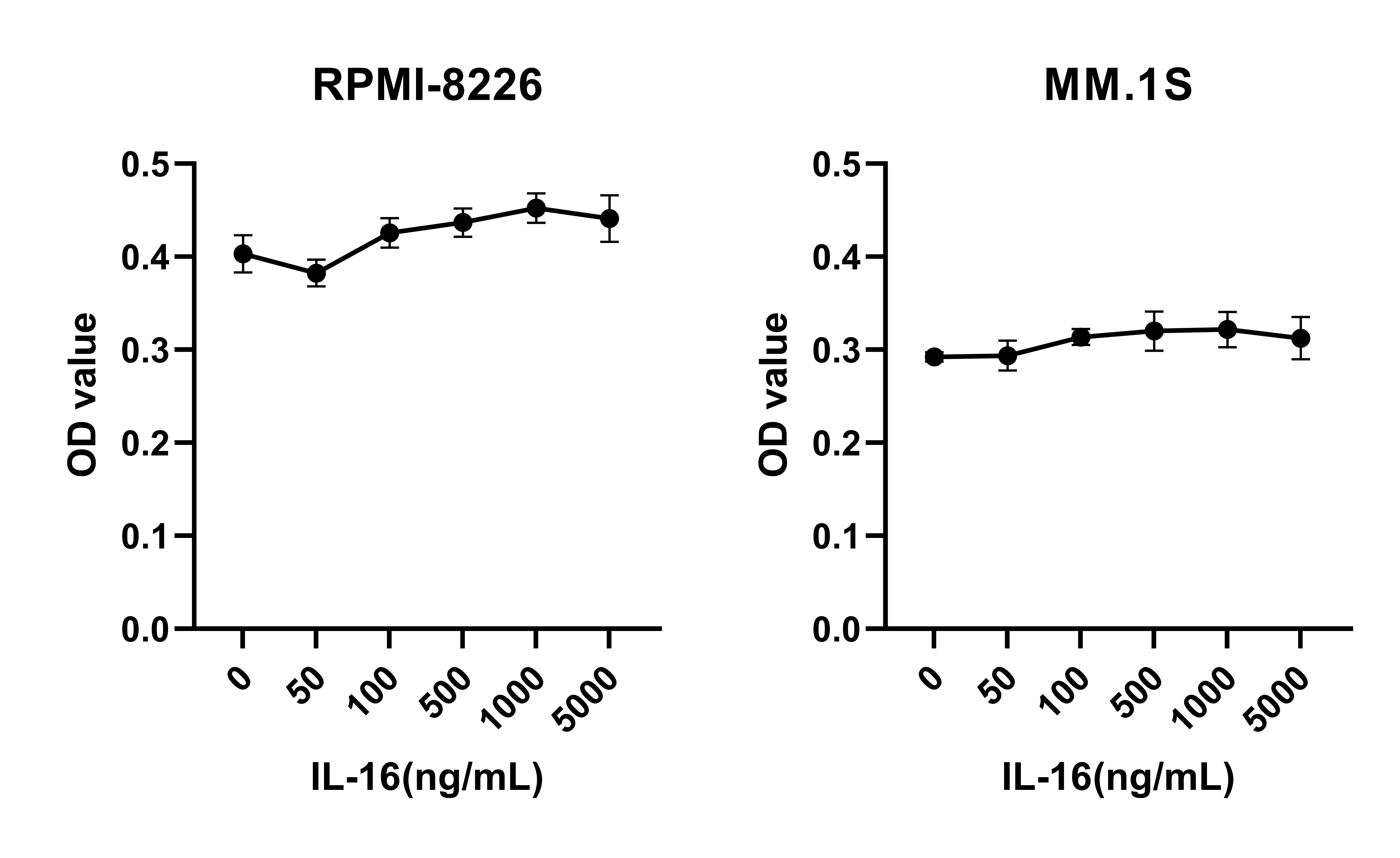

Supplement: Supplementary file 1 — Figure S1: cam471651‐sup‐0001‐FigureS1.jpg. [file CAM4-15-e71651-s003.jpg]

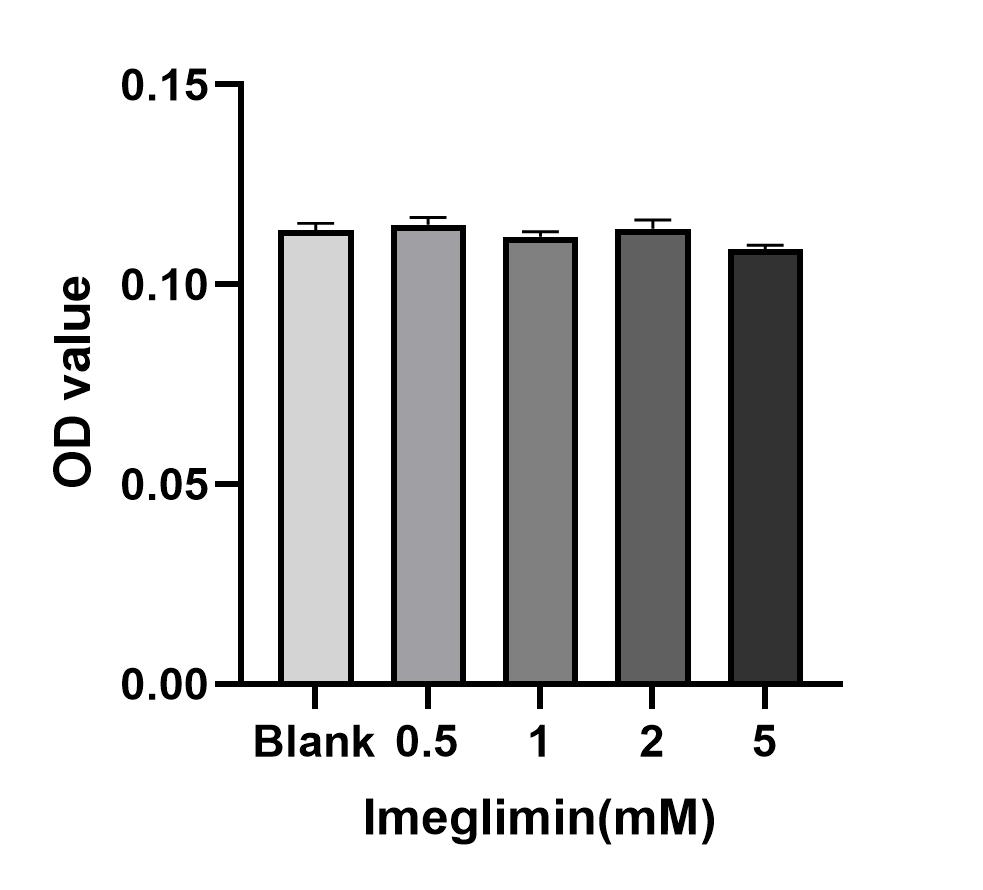

Supplement: Supplementary file 2 — Figure S2: cam471651‐sup‐0002‐FigureS2.jpg. [file CAM4-15-e71651-s002.jpg]

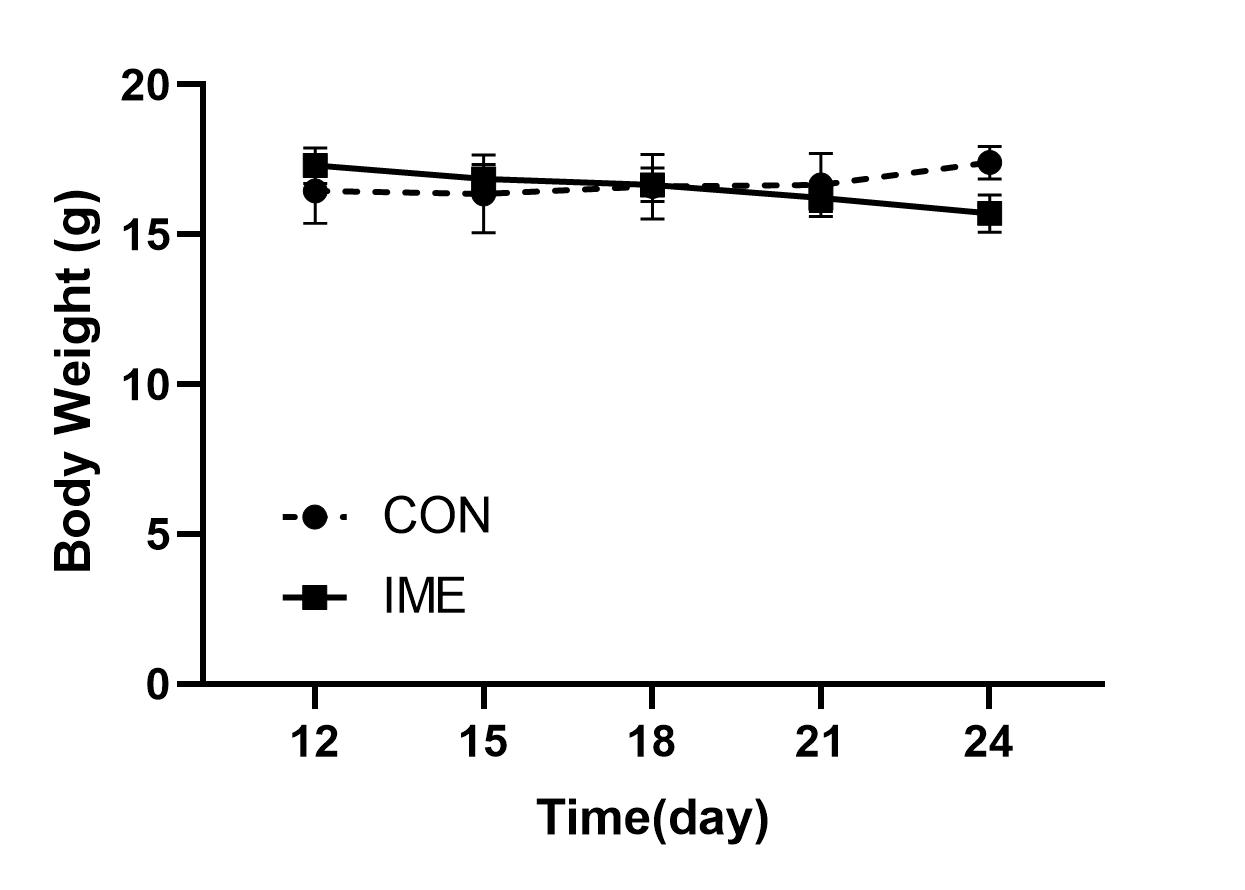

Supplement: Supplementary file 3 — Figure S3: cam471651‐sup‐0003‐FigureS3.jpg. [file CAM4-15-e71651-s004.jpg]

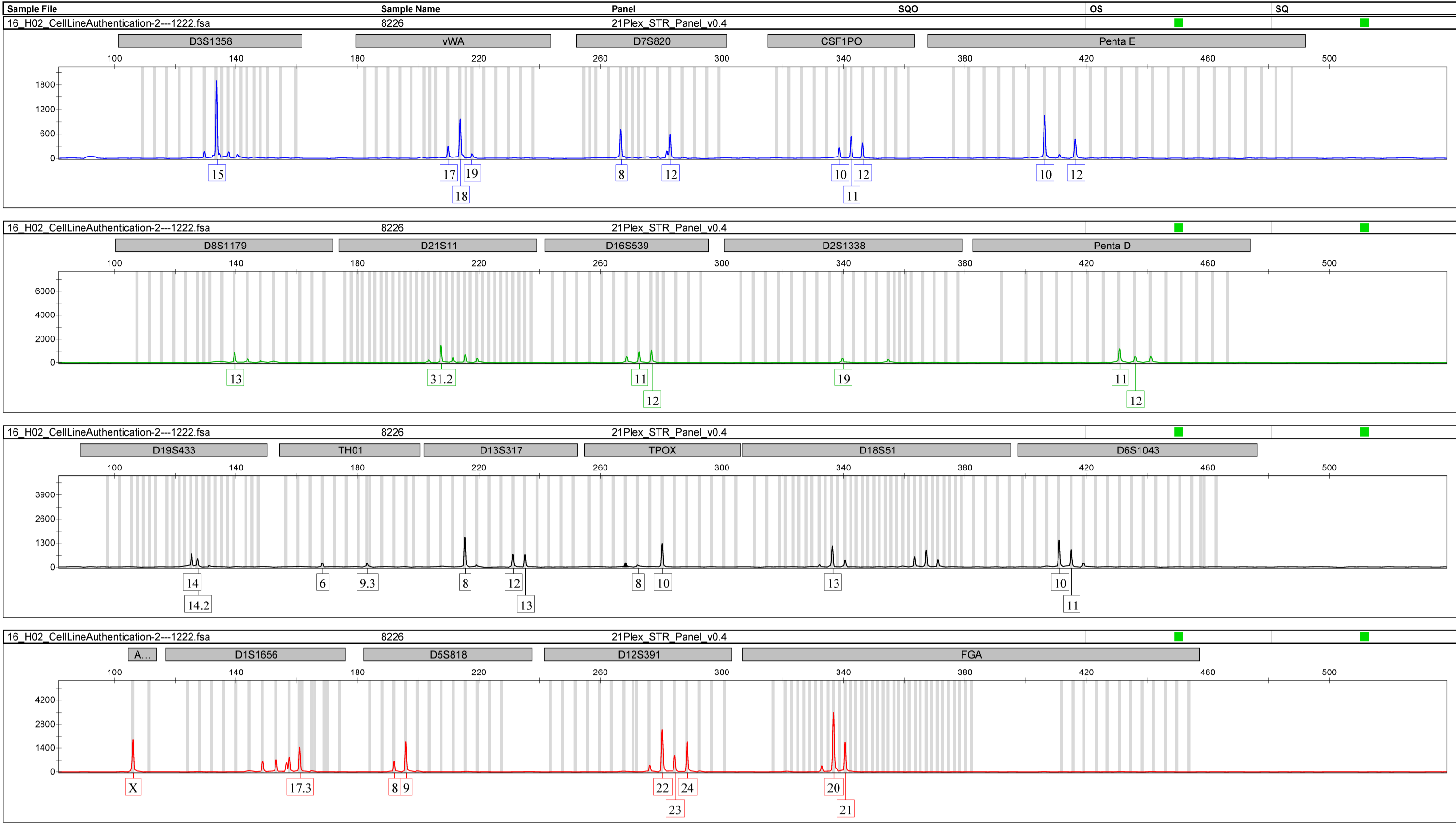

Supplement: Supplementary file 4 — Data S1: cam471651‐sup‐0004‐Supinfo.zip. [file CAM4-15-e71651-s001.zip › cam471651-sup-0004-Supinfo.pdf]

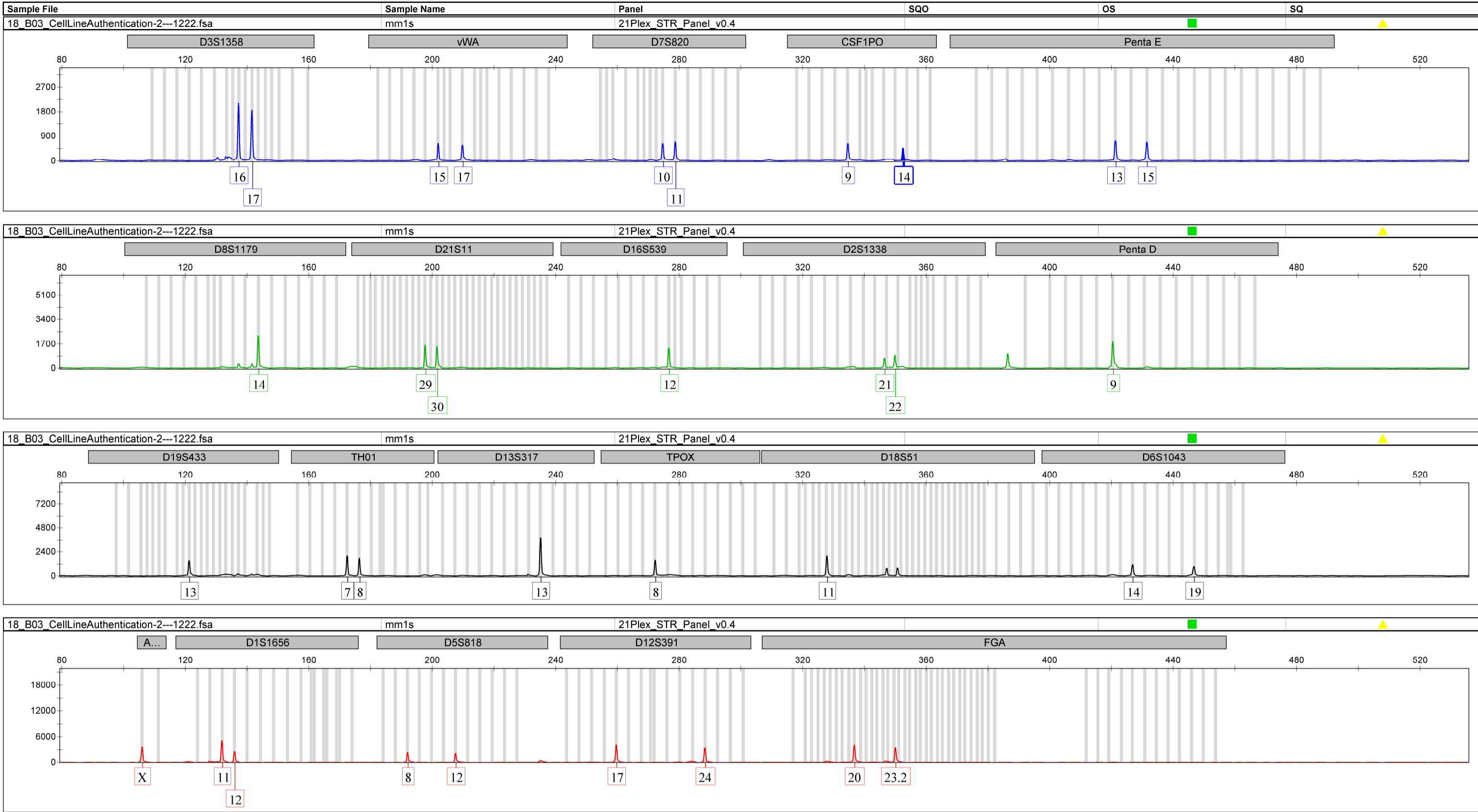

Supplement: Supplementary file 4 — Data S1: cam471651‐sup‐0004‐Supinfo.zip. [file CAM4-15-e71651-s001.zip › cam471651-sup-0005-Supinfo1.pdf]

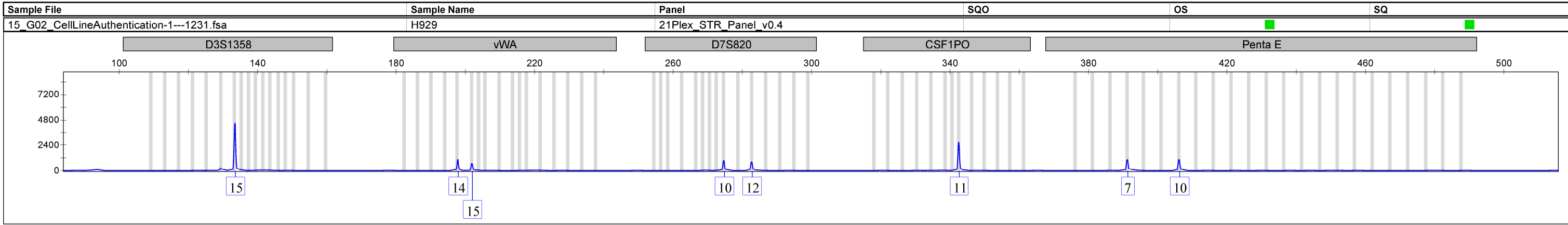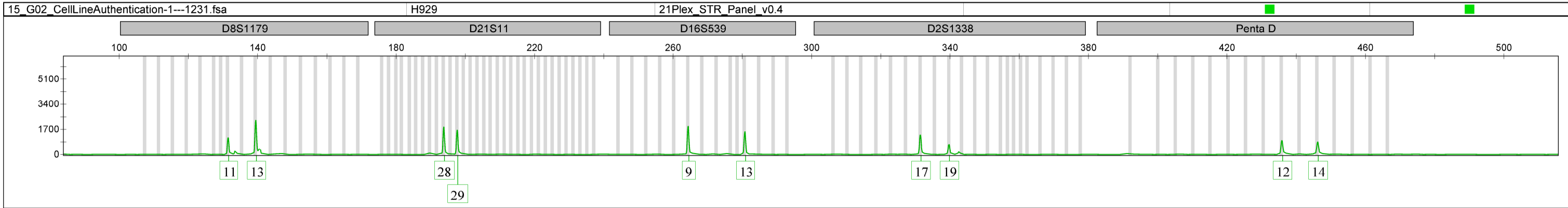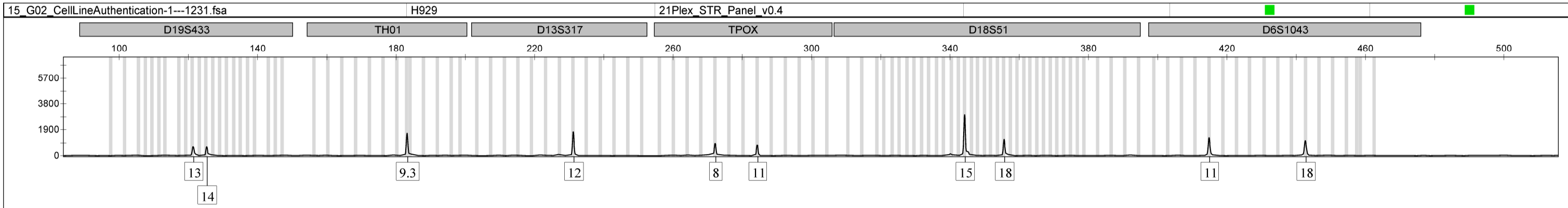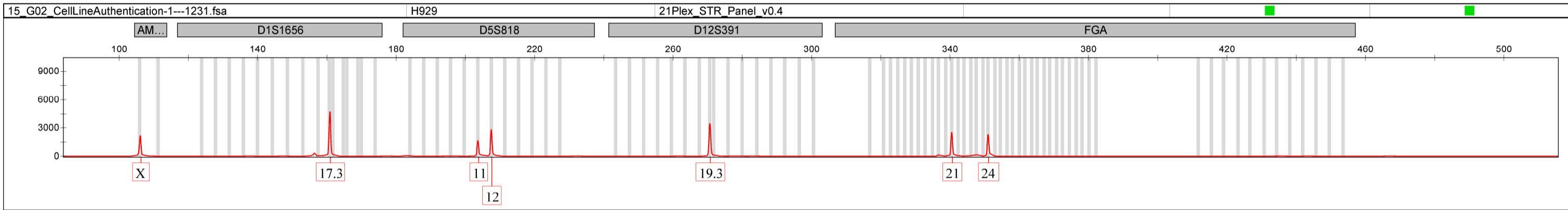

Supplement: Supplementary file 4 — Data S1: cam471651‐sup‐0004‐Supinfo.zip. [file CAM4-15-e71651-s001.zip › cam471651-sup-0006-Supinfo2.pdf]
